# Fucose supplementation as a novel treatment approach for GLUT1 deficiency syndrome

**DOI:** 10.1007/s00415-026-14211-3

**Published:** 2026-10-15

**Authors:** Julia Moomann, Manfred Fobker, Clemens Gardemann, Petra Schulze Westhoff, Hudson H. Freeze, Bobby G. Ng, Oliver Schwartz, Otfried Debus, Pankaj K. Singh, Matthieu G. Colpaert, Giancarlo G. Valdivia, Olivia D’Agati, Matthew S. Gentry, Julien Park, Thorsten Marquardt

**Affiliations:** 1 Department of General Paediatrics, University Hospital Münster, Münster, Germany; 2 Centre of Laboratory Medicine, University Hospital Münster, Münster, Germany; 3 Sanford Children’s Health Research Center, Sanford Burnham Prebys Medical Discovery Institute, La Jolla, CA USA; 4 Clemenshospital, Department of Paediatrics, Münster, Germany; 5 Department of Biochemistry and Molecular Biology, College of Medicine, University of Florida, Gainesville, FL USA; 6 Center for Advanced Spatial Biomolecule Research, University of Florida, Gainesville, FL USA

**Keywords:** Fucose, GLUT1 deficiency syndrome, Hypoglycorrhachia, Ketogenic diet

Sirs,

Glucose transporter type 1 (GLUT1), encoded by *SLC2A1*, is a facilitative uniporter mediating the transport of glucose across the plasma membrane of mammalian cells. It is highly expressed at the blood–brain barrier (BBB), where it constitutes the principal route for glucose entry into the central nervous system, and is therefore essential for cerebral energy metabolism [1].

Haploinsufficiency of *SLC2A1* causes GLUT1 deficiency syndrome (GLUT1DS, MIM # 606,777), an autosomal dominant disorder of brain energy metabolism [2]. Because GLUT1 is the near-exclusive route for glucose entry into the brain across the BBB, reduced transporter function limits neural glucose availability, causing a chronic cerebral energy deficit that impairs brain development and function [3]. Patients classically present with infantile-onset epilepsy, deceleration of head growth, developmental delay, and complex, often paroxysmal movement disorders; symptoms evolve in an age-specific pattern, with ataxia and paroxysmal exertion-induced dyskinesia becoming prominent in adolescence and adulthood [4]. Hypoglycorrhachia is a biochemical hallmark [5]. A subset of patients with GLUT1 deficiency also displays a haematological phenotype, comprising mild haemolytic anaemia with echinocytic (stomatin-associated) erythrocyte deformation and altered red-cell cation content (increased sodium, decreased potassium). Because the erythrocyte relies on GLUT1 as its principal glucose transporter, impaired glucose uptake compromises the energy supply required to maintain cation homeostasis; the resulting cation leak causes red cells to swell and undergo haemolysis [6].

The mainstay of treatment is a ketogenic diet, which supplies ketone bodies as an alternative fuel for the brain, thereby bypassing the defective glucose transport [5]. Early initiation can improve seizure control and support cognitive development. However, the response is incomplete in a substantial subgroup of patients—particularly with respect to movement disorders and cognitive outcome—underscoring the need for complementary therapeutic strategies [7].

Recent evidence indicates that GLUT1 also functions as a plasma-membrane fucose transporter, linking it to protein fucosylation in addition to its canonical role in glucose uptake [8]. This is mechanistically significant because deficient fucosylation produces a recognisable clinical phenotype: in SLC35C1-CDG (MIM #266,265), loss of the GDP-fucose transporter causes a multisystem disorder with growth restriction, intellectual disability, and recurrent infections [9, 10], which responds substantially to oral fucose therapy [11].

In this report, we identified substantial hypofucosylation in a patient with GLUT1 deficiency. Oral fucose supplementation was associated with both clinical and biochemical improvement, suggesting that defective fucosylation contributes to the GLUT1DS phenotype and may represent a tractable therapeutic target.

The patient is a boy, currently 3 years 8 months, born at 34+4 weeks. The neonatal course included bilateral congenital cataracts (surgically corrected at one month), a parieto-occipital haemorrhage with intraventricular extension despite normal coagulation, and haemolytic anaemia requiring transfusions. Genetic testing identified a heterozygous de novo *SLC2A1* variant (c.1306_1308del; p.Ile436del) (Fig. 1A). CSF analyses gave glucose 1.96 mmol/l with CSF-to-serum ratios of 0.34–0.53, consistent with impaired BBB glucose transport. The diagnosis of GLUT1DS was made at the age of two months. Interictal EEG showed right posterior/temporo-occipital focal epileptiform discharges concordant with the haemorrhage site (Fig. 1C); no seizures were captured. Development was severely delayed (motor level ~3 months at 16 months; GMFCS V). A ketogenic diet (3:1 escalated to 4:1) with exogenous ketone ester and sodium citrate supplementation was initiated at two months and maintained β-hydroxybutyrate at 2–8 mmol/l (Fig. 1B); the child remained seizure-free but showed no developmental progress despite sustained ketosis.

**Fig. 1:**
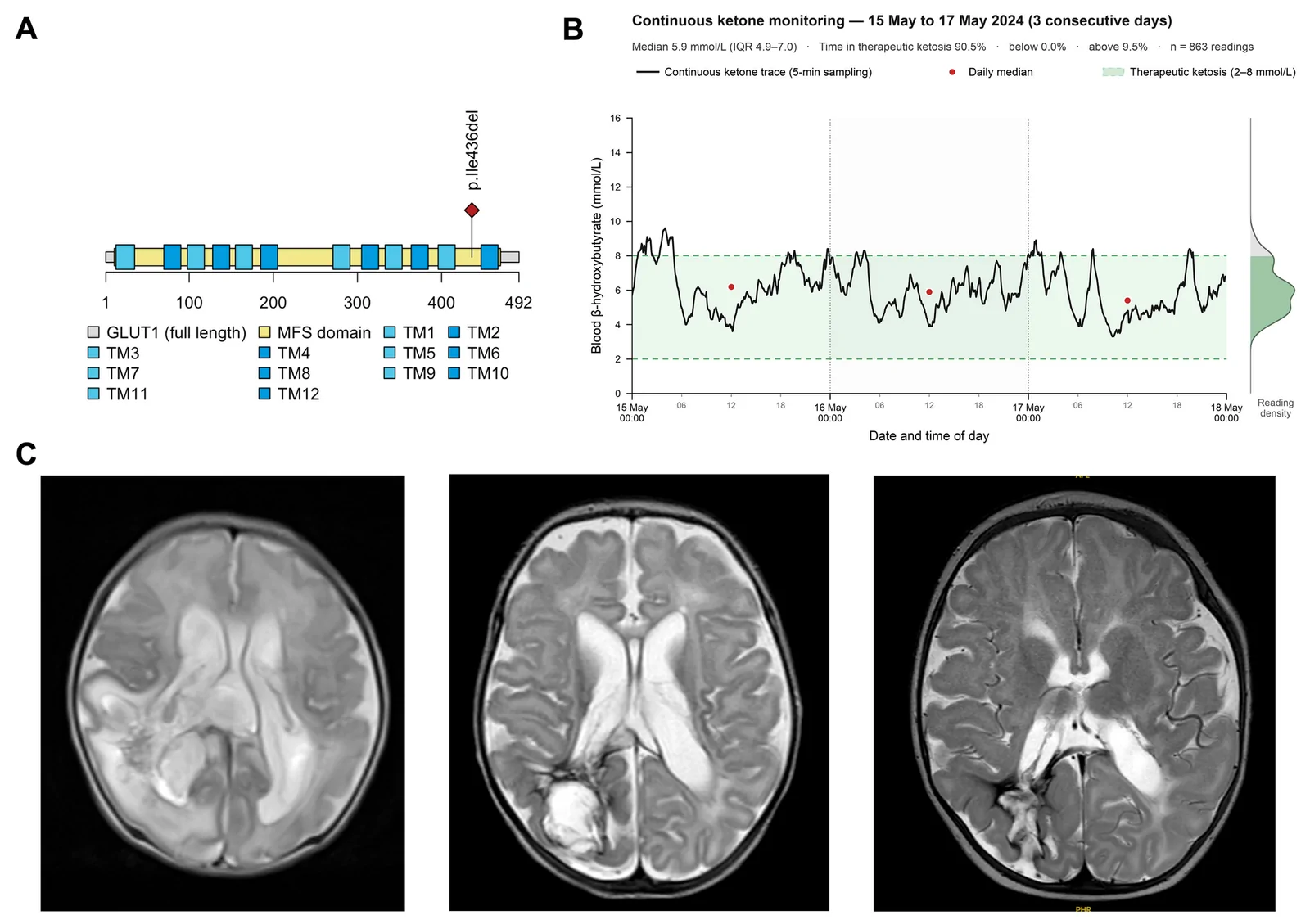
Molecular, metabolic and neuroimaging characterisation of a patient with GLUT1 deficiency syndrome. (A) Schematic of the GLUT1 protein (encoded by *SLC2A1*; 492 amino acids) showing the major facilitator superfamily (MFS) domain and 12 transmembrane helices (TM1–TM12), with the position of the identified p.Ile436del variant (red diamond). (B) Continuous ketone monitoring over three consecutive days (15–17 May 2024) during ketogenic dietary therapy. Blood β-hydroxybutyrate trace (5-min sampling; n = 863) with daily medians (red circles) and target therapeutic range (2–8 mmol/L, green shading). Median 5.9 mmol/L (IQR 4.9–7.0); 90.5% of time within range. Reading density shown at right. (C) Serial axial T2-weighted MRI at 6 days, 7 weeks and 6 months of age (left to right), demonstrating delayed myelination, mildly prominent lateral ventricles and diffuse volume loss, without focal structural lesion

L-Fucose was initiated at the age of 22 months at 1 g/day (≈0.10 g/kg/day) and escalated to 5 g/day within four days, then to 0.7 g/kg/day by day 90, 1 g/kg/day by ~day 240, and 1.5 g/kg/day from day 480. Mannose (day 300, 1→10 g/day) was discontinued after two months due to diarrhoea. Treatment was an individual treatment trial under German law (Einzelheilversuch), exempt from IRB approval per the Ethik-Kommission Westfalen-Lippe, with consent by both parents per the Declaration of Helsinki (2013).

CSF L-fucose was measured by UHPLC. Glycoprotein fucosylation was assessed by AAL-lectin blotting (covering α-1,2/1,3/1,4/1,6 linkages); β-trace protein was immunoprecipitated from CSF (protein A sepharose) followed by AAL staining or immunoblotting. Metabolomics of erythrocytes, plasma, and CSF used GC–MS. Full methods are in the Supplementary material. Analyses used R v4.6.0; controls are median with IQR (n = 7 CSF). Pearson's correlation related fucose dose to CSF fucose (first four paired measurements). Lectin intensity was normalised to total protein and mean control (% control; n = 3), compared by one-way ANOVA with Tukey's test in GraphPad Prism v11.0.2. Figures were created in part with biorender.com.

CSF free fucose was below the low-normal range (0.48 mg/l; reference 0.6–0.84), corroborated by GC–MS showing reduced free fucose in erythrocytes, plasma, and CSF (Fig. 2 A-D). AAL binding was significantly reduced relative to controls in erythrocyte lysates (81.4 ± 1.75%, P = 0.04), plasma (69.0 ± 3.52%, P < 0.001), and CSF (62.8 ± 3.96%, P = 0.007) (Fig. 2 E–G). Because most CSF proteins are blood-derived, we examined the predominantly CNS-produced β-trace protein [12]: its fucosylation (61.3 ± 5.29%, P = 0.002) and abundance (20.4 ± 2.66%, P < 0.001) were both reduced, indicating the defect reaches CNS-derived proteins (Fig. 2H, Fig. S1). The pre-supplementation erythrocyte profile also showed reduced glucose and citrate with elevated lactate, glycine, and creatinine (Fig. 2I).

**Fig. 2:**
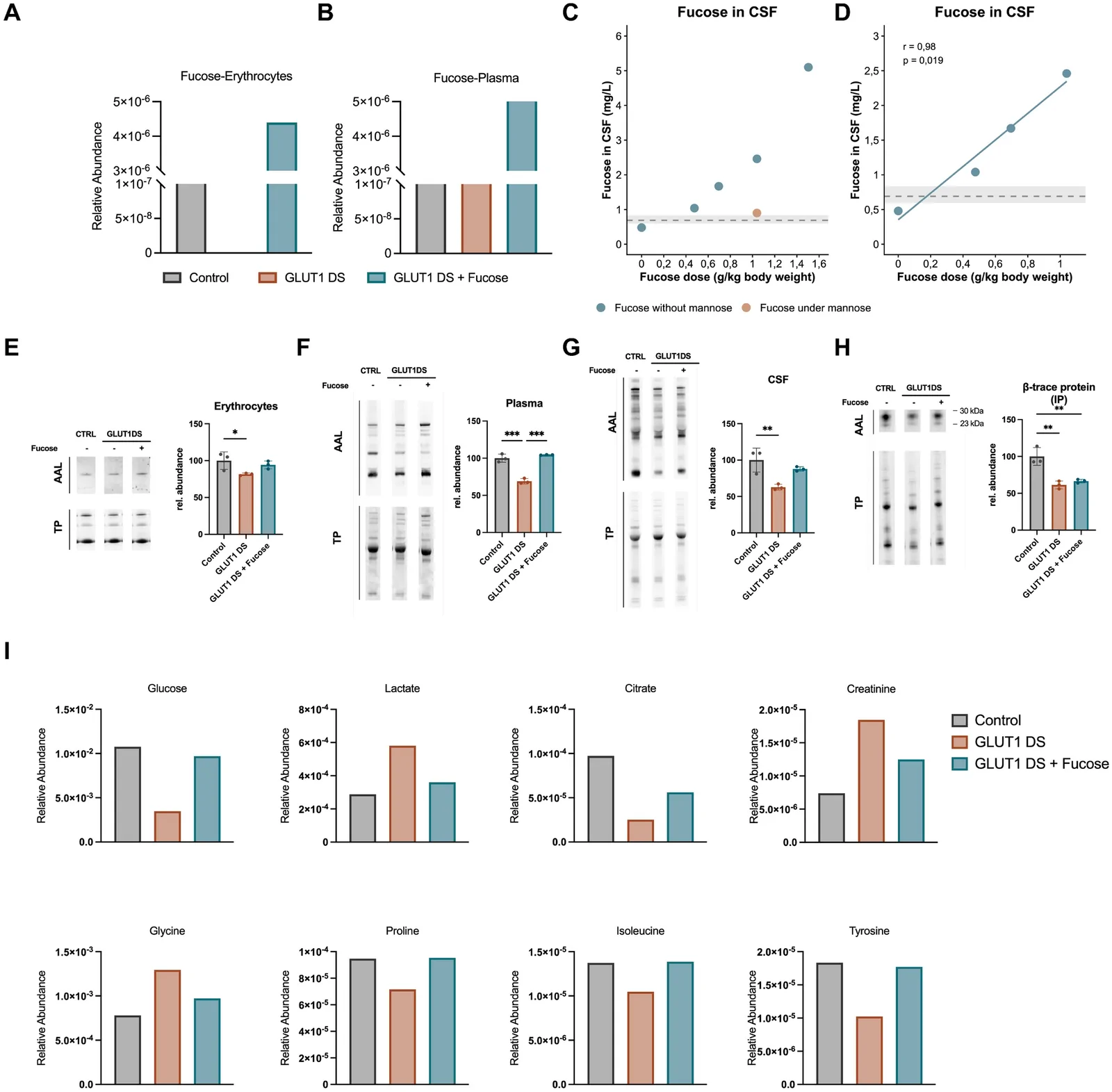
GLUT1DS is associated with systemic and central hypofucosylation that is largely restored by oral L-fucose, with the notable exception of the CNS-derived β-trace protein. Free fucose in the polar metabolite fraction, quantified by GC–MS, is shown for (A) erythrocytes and (B) plasma in a healthy control (grey) and in the GLUT1DS patient before (orange) and after (teal) L-fucose therapy. (C) HPLC-based CSF fucose concentration, which lay below the paediatric reference range (0.6–0.84 mg/L; interquartile range of children aged 1–5 years, n = 7) at baseline and rose above control levels during therapy. (D) Pearson correlation between administered L-fucose dose and CSF fucose concentration, showing a strong positive linear relationship (r = 0.98, P = 0.019) across the first four paired measurements, prior to mannose treatment. Protein fucosylation was assessed by quantitative Aleuria aurantia lectin (AAL) blotting, with AAL signal normalised to total protein (TP), in control (CTRL), GLUT1DS, and fucose-supplemented GLUT1DS (GLUT1DS + Fucose) samples: relative abundance of AAL-reactive glycans is shown for (E) erythrocytes, (F) plasma, and (G) total CSF, each with representative AAL and TP blots. (H) Immunoprecipitated (IP) β-trace protein, showing AAL-based fucosylation normalised to TP with representative blots; β-trace fucosylation remained reduced despite systemic correction. (I) Selected erythrocyte metabolites showing partial restoration towards the control profile following L-fucose therapy. AAL and immunoblot data (E–H) are presented as mean ± SD of three technical replicates; GC–MS and HPLC data (A–D, I) derive from single control and patient samples (n = 1 per condition). Statistical significance was determined by ordinary one-way ANOVA with Tukey’s post-hoc test (*P < 0.05; **P < 0.01; ***P < 0.001)

Supplementation raised fucose above control levels across all compartments (Fig. 2 A–D), with CSF fucose correlating linearly with dose (r = 0.98, *P* = 0.019). Global protein fucosylation normalised in erythrocytes, plasma, and total CSF (Fig. 2E-G). In contrast, β-trace protein fucosylation did not increase significantly (66.5 ± 2.48% vs 61.3 ± 5.29%, *P* = 0.70, Fig. 2H), though its abundance rose (28.99 ± 1.64% vs 20.4 ± 2.66%, *P* = 0.05, Fig. S1). Mannose produced no change. Following therapy, several erythrocyte metabolites (glucose, citrate, lactate, amino acids) shifted towards control, while others did not (Fig. 2I, Fig. S2); plasma showed a similarly selective response (Fig. S3), and CSF metabolites remained largely unchanged despite increased CSF fucose (Fig. S4). Thus the response was metabolite- and compartment-specific.

Supplementation began at 22 months with the concurrent regimen unchanged. Within two months, parents and physicians consistently reported accelerated development: acquisition of rolling, improved head control, and increased social interaction and reciprocal play, with continued gradual gains thereafter, resulting in an improve GMFCS level of IV. Visual status remained stable. Serial EEG showed persistence of right focal discharges in February 2025 but resolution of the focal discharges and lateralisation by October 2025 (~ one year on). The patient remained seizure-free, and L-fucose was well tolerated over 18 months with no adverse effects.

## Discussion

This study provides the first evidence that GLUT1DS is accompanied by a defect in fucose availability and fucosylation in humans, extending the phenotype to include systemic and central hypofucosylation. This is mechanistically coherent with the recent identification of GLUT1 as a highly efficient L-fucose transporter operating in the physiological micromolar range, non-competitively with millimolar glucose [8]; defective GLUT1-mediated fucose transport across the BBB plausibly underlies the reduced CSF fucose and CNS protein hypofucosylation observed.

Reduced fucosylation of the predominantly CNS-produced β-trace protein [12] indicates the defect is not merely a reflection of peripheral hypofucosylation but affects proteins made by CNS-resident cells, localising part of the defect to the brain compartment, consistent with GLUT1's role at the BBB [13].Oral L-fucose raised fucose across all compartments, with CSF fucose correlating linearly with dose, and normalised global fucosylation in erythrocytes, plasma, and total CSF—biologically plausible given salvage-pathway incorporation of exogenous fucose [14] and consistent with responses reported in SLC35C1-CDG [11].

A notable dissociation emerged: global, largely blood-derived CSF fucosylation normalised, whereas CNS-derived β-trace protein fucosylation did not (Fig. 2G, H). This may indicate that supplemented fucose reaches the CSF but incompletely accesses the fucosylation machinery of CNS-resident cells, consistent with evidence that GDP-fucose exists in multiple, differentially accessible pools that respond unequally to exogenous fucose [14]. Alternatively, β-trace protein may not represent overall CNS fucosylation, a possibility supported by the observed clinical improvement. Direct brain-tissue measurement will be required to distinguish these.

These findings carry several implications. Hypofucosylation may be a previously unrecognised component of GLUT1DS pathophysiology contributing to the neurological phenotype, given the importance of fucosylation in the CNS [15]. AAL binding and fucosylation may serve as candidate biomarkers complementing established diagnostics. Oral L-fucose is positioned as a safe candidate adjunctive therapy for systemic hypofucosylation, with central efficacy yet to be established. The main limitation is the single-patient design, requiring cohort validation; persistent β-trace hypofucosylation raises questions about the routes and efficiency of CNS fucose delivery, and future work should test whether higher doses or alternative delivery achieve CNS correction and clinical benefit.

In summary, GLUT1DS is associated with hypofucosylation extending to CNS-derived proteins; oral L-fucose corrects peripheral and global CSF fucosylation while leaving CNS-intrinsic fucosylation largely uncorrected, accompanied by partial, compartment-specific metabolic restoration—opening a new avenue for understanding and treating GLUT1DS. Currently ongoing trials of L-fucose supplementation in GLUT1DS on a larger scale (NCT07432490) will doubtlessly inform future treatment approaches for this devastating disorder.

## Supplementary Information

Below is the link to the electronic supplementary material.

Supplementary file1 (PDF 8745 KB)

## Data Availability

De-identified clinical and genetic data related to this study can be shared upon reasonable request. Interested researchers may contact the corresponding authors to discuss data access in accordance with participant consent and institutional guidelines.
