# Supplementary material for: Fucose supplementation as a novel treatment approach for GLUT1 deficiency syndrome
Source: J Neurol. 2026 Oct 15;273(11):663. doi: 10.1007/s00415-026-14211-3 (PMC13672255; doi:10.1007/s00415-026-14211-3)
Supplement: Supplementary file 1 [file 415_2026_14211_MOESM1_ESM.pdf]

Fucose supplementation as a novel treatment approach for GLUT1 deficiency syndrome

— Supplemental Material —

Julia Moomann[1], Manfred Fobker[2], Clemens Gardemann[1], Petra Schulze Westhoff[1], Hudson H. Freeze[3], Bobby Ng[3], Oliver Schwartz[1], Otfried Debus[4], Pankaj K. Singh[5], Matthieu G. Colpaert[5], Giancarlo G. Valdivia[5], Olivia D'Agati[5], Matthew S. Gentry[5,6], Julien Park[1,†] & Thorsten Marquardt[1,†]

Author affiliations:

[1] University Hospital Münster, Department of General Paediatrics, Münster, Germany

[2] University Hospital Münster, Centre of Laboratory Medicine, Münster, Germany

[3] Sanford Childrens Health Research Center, Sanford Burnham Prebys Medical Discovery Institute, La Jolla, CA, USA.

[4] Clemenshospital, Department of Paediatrics, Münster, Germany

[5] Department of Biochemistry & Molecular Biology, College of Medicine, University of Florida, Gainesville, FL, USA

[6] Center for Advanced Spatial Biomolecule Research, University of Florida, Gainesville, FL, USA

† These authors contributed equally

1. Supplemental Methods

Fucose quantification by High-performance liquid chromatography (HPLC) in CSF

L-fucose, $^{13}C_2$-fucose (LGC Standards, Rotherham, UK), D-mannose and $^{13}C_6$-mannose (internal standard, IS), acetonitrile, water and formic acid (LC-MS grade) were provided by Merck, Darmstadt, Germany. Stock solution of analytes and internal standard (each 50 mg/L) were stored at -20°C in water. The calibration curve for fucose was prepared by serial dilution with water (0.1, 1, 5, 50 mg/L) and for mannose 10-fold concentrations were used.

50 µl of sample were thoroughly mixed with -20°C cold 150 µl acetonitrile (+ 5mg/L IS) and centrifuged at 12000 rpm for 5 min to precipitate proteins. 100 µl of the supernatant was transferred into an autosampler tube and 5 µl was injected into the LC/MS/MS system.

Liquid chromatography was performed on a Shimadzu Nexera XR UHPLC system coupled to an LCMS-8050 triple quadrupole mass spectrometer. Isocratic separation was achieved at 50 °C on a HILIC column (MassChrom® Aminoacid, 75100, Chromsystems, Munich, Germany) using acetonitrile and 15% 0.1% formic acid in water (v/v) at a flow rate of 0.8 ml/min. The MS operated in ESI negative mode with a nebulizing gas flow of 3 L/min; heating gas flow 15 L/min; drying gas flow 5 L/min; interface temperature 300 °C; DL (desolvation line) temperature 250 °C and heat block temperature 400 °C. The optimized transitions were [m/z 209.2 → 163.2] for fucose, [m/z 225.1 → 179.1] for mannose and m/z 185 → 92 for IS (CE: 10 eV), utilizing a dwell time of 50 ms. Retention time for fucose was 3.1 min and 4.7 min for mannose. Data were processed via LabSolutions software (Version 5.89, Shimadzu).

Lectin blotting

Fucosylation of glycoproteins was assessed by AAL-Lectin blotting, thus covering Fucose bound in α-1,2, α-1,3, α-1,4, and α-1,6 linkages.[36] Interfering haemoglobin was removed from erythrocyte samples using HemogloBind™ (#H0145-05, Biotech Support Group, Monmouth Junction, NJ, USA) according to the manufacturer's protocol. Protein concentration was

determined using a BCA assay (#71285-3, Merck Millipore, Darmstadt, Germany) and adjusted to 1 µg/µl with Tris lysis buffer (50 mM Tris, 150 mM NaCl, 1% w/v Triton X-100, protease inhibitor [#04693159001, Roche, Mannheim, Germany]).

NuPAGE 4–12% Bis-Tris gels (#NP0321PK2, Thermo Fisher Scientific, Waltham, MA, USA) were loaded with 5 µg (plasma) or 10 µg of protein (CSF, erythrocyte lysates). Following transfer onto 0.45 µm nitrocellulose membranes (#10600002, Amersham, Cytiva Europe GmbH, Freiburg, Germany), membranes were stained with Revert 700 Total Protein Stain (#926-11010, LI-COR Biosciences, Lincoln, NE, USA) according to the manufacturer's protocol for subsequent normalization and visualization.

Membranes were blocked with Carbo-Free Blocking Solution (#SP-5040, Vector Laboratories, Newark, CA, USA) for 30 minutes and then incubated with biotinylated AAL (#B-1395-1, Vector Laboratories, Newark, CA, USA) at a 1:1000 dilution in PBS (phosphate-buffered saline) containing 0.1 mM $CaCl_2$ for one hour. Membranes were washed five times for five minutes each in 0.05% PBS-Tween. Membranes were subsequently incubated with the secondary detection reagent Streptavidin-IRDye 800CW at a 1:5000 dilution in 0.15% PBS-Tween containing 0.01% SDS for one hour. Following three washes of five minutes each in PBST, blots were visualized on a ChemiDoc Imaging System (Bio-Rad, Hercules, CA, USA). Fluorescence signal was normalized to total protein.

Immunoprecipitation of β-trace protein

β-trace protein was immunoprecipitated from cerebrospinal fluid (CSF) as follows. Per reaction, 150 µL of CSF was combined with 3 µL of rabbit anti-β-trace antibody (#10754-2-AP, ProteinTech, Rosemont, IL, USA), 3 µL of protease inhibitor cocktail (#ab271306, Abcam, Cambridge, UK), and 70 µL of Protein A-Sepharose (10% suspension; #P3391, Merck Millipore, Darmstadt, Germany). Samples were incubated overnight at room temperature under continuous rotation to allow formation of antibody–antigen–Protein A complexes.

The Sepharose beads were subsequently washed three times with PBS, with the supernatant removed by aspiration after each wash. Immunoprecipitated proteins were eluted by adding 40 µL

of 1 x Licor bio sample buffer (#928-40004, LI-COR Biosciences, Lincoln, NE, USA) containing 10% mercaptoethanol, followed by brief vortexing and denaturation for 10 minutes at 99 °C. Samples were then centrifuged briefly to pellet the beads, and the supernatant was collected for downstream analysis. Eluates were resolved by SDS-PAGE and transferred to nitrocellulose membranes as described above. Fucosylation of immunoprecipitated β-trace protein was first assessed by AAL lectin blotting (as detailed above).

Immunoblotting of β-trace protein

The same membrane was subsequently probed by conventional immunoblotting to confirm the identity and loading of β-trace protein. Following blocking, membranes were incubated with anti-β-trace primary antibody (see above) at a dilution of 1:1000 in PBST containing 1% bovine serum albumin (BSA) overnight at 4°C. After washing 3 x for 10 min in PBST, membranes were incubated with VRDye 490 Goat Anti Rabbit IGG Secondary Antibody (#926-49020, LI-COR) at a dilution of 1:5000 in PBST containing 1% BSA for 1h. Following three 10 min washes in PBST, membranes were visualized on a ChemiDoc Imaging System (Bio-Rad), and image analysis was performed using Image Lab software (Bio-Rad).

Gas Chromatography-Mass Spectrometry (GC-MS) Metabolomics

Erythrocyte pellets, plasma, and CSF (30 µL plasma or CSF) were extracted in 1 mL of ice-cold 50% methanol containing 1 µL of 20 mM L-norvaline (Merck, N7627), vortexed, incubated on ice for 30 min, and centrifuged at 15,000 rpm for 10 min at 4°C to separate the polar metabolite-containing supernatant from the protein/lipid pellet. The polar fraction was snap-frozen in liquid nitrogen and stored at −80°C. The protein pellet was hydrolysed in 100 µL of 6 N HCl at 95°C for 2 h, quenched with 200 µL of 100% methanol containing 200 µM L-norvaline, vortexed, and centrifuged (15,000 rpm, 10 min, 4°C). Aliquots of the polar (250 µL) and protein hydrolysate (300 µL) fractions were dried under vacuum (10^{-3} mbar) and derivatized with 20 mg/mL methoxyamine hydrochloride in pyridine (1.5 h, 30°C), followed by N-methyl-N-(trimethylsilyl)trifluoroacetamide (MSTFA) for 30 min at 37°C. Samples (2 µL, 1:5 split injection)

were analysed on an Agilent 7800B gas chromatograph coupled to a 5977B single quadrupole mass spectrometer. The GC oven was held at 60°C for 1 min, ramped to 325°C at 10°C/min, and held for 10 min. Mass spectra were acquired in EI mode (70 eV; m/z 50–550) with transfer line and source temperatures of 290°C and 280°C, respectively. Metabolites were identified by matching retention times and EI fragmentation patterns against the Agilent Fiehn 2013 Metabolomics RTL Database using AMDIS software. Relative metabolite abundances were quantified using the Data Extraction for Stable Isotope-labelled Metabolites (DEXSI) software. Polar metabolites from erythrocytes were normalized to total protein abundance determined from amino acid signals, whereas plasma and CSF metabolites were normalized to the L-norvaline internal standard.

2. Figure S 1

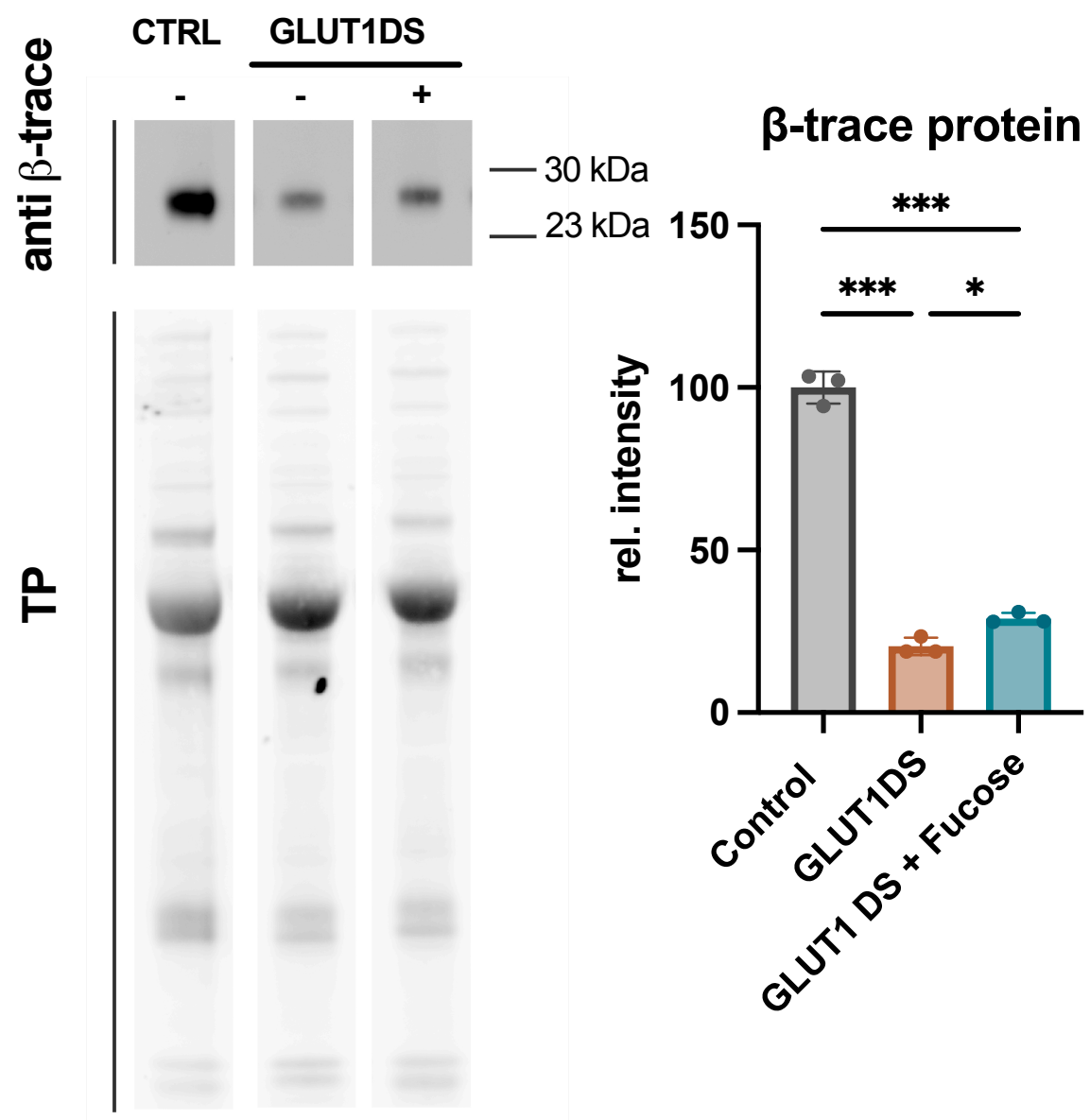

Figure S 1 - Immunoblot against β-trace protein (anti β-trace), with band intensity normalized to TP and quantification of relative intensity. Data are presented as mean ± SD of three technical replicates, with representative blots shown. Statistical significance was determined by ordinary one-way ANOVA with Tukey's post-hoc test (*P < 0.05; **P < 0.01; ***P < 0.001).

3. Figure S 2

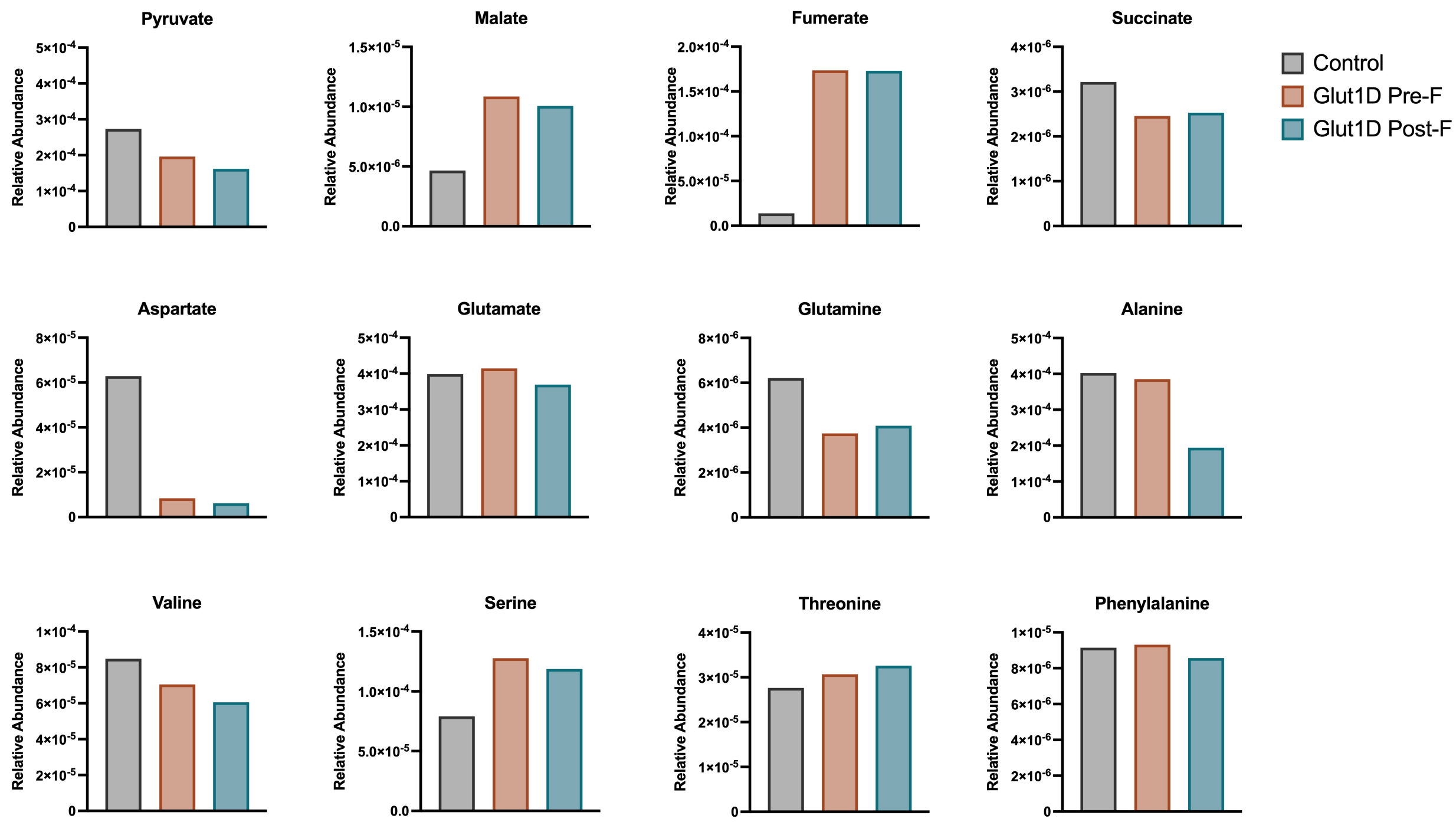

Figure S2 - GC-MS-based metabolomic analysis of erythrocytes. Bar graphs show the relative abundance of metabolites in the healthy control (gray), and a GLUT1DS patient before (orange) and after (teal) L-fucose therapy (**n = 1**).

4. Figure S 3

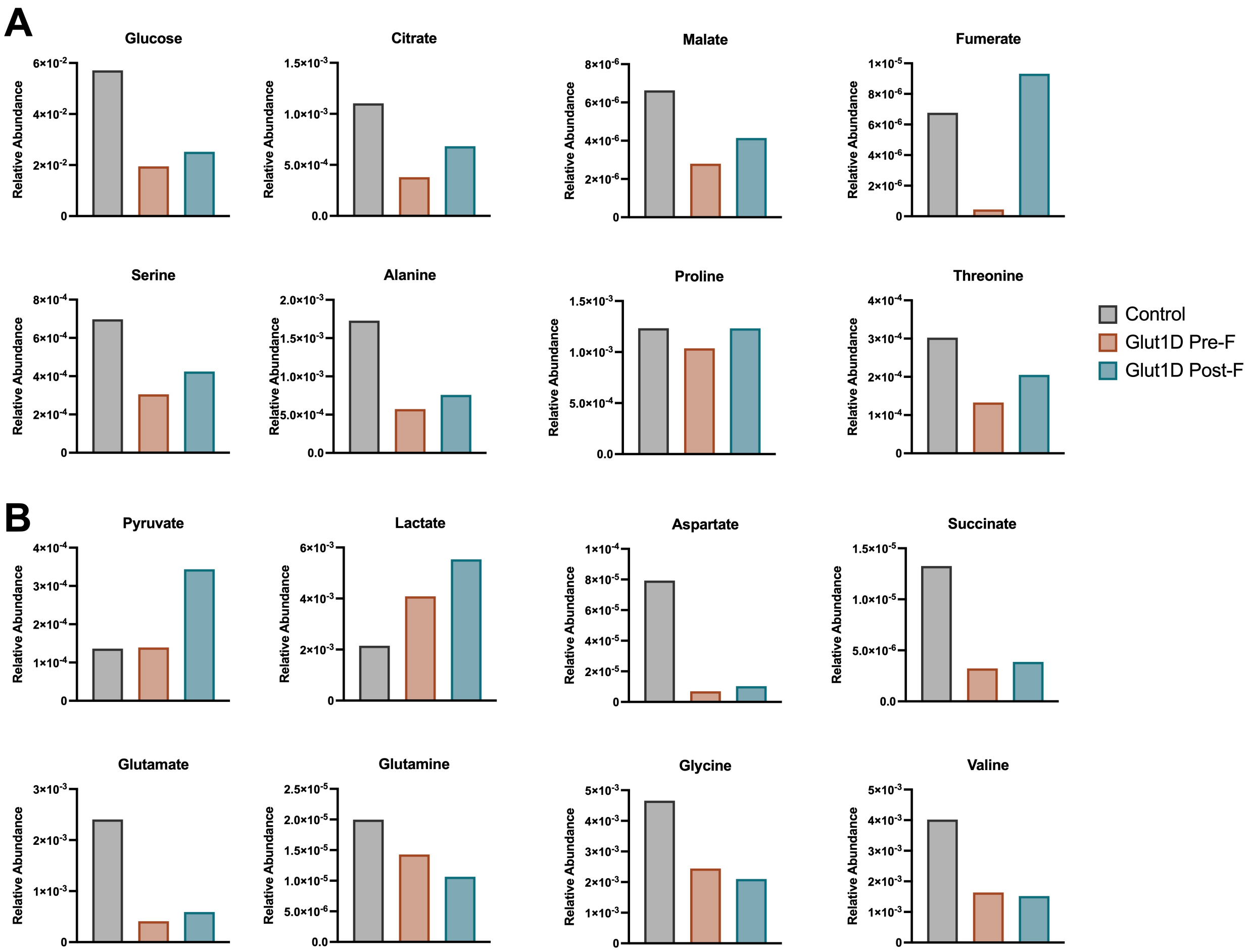

Figure S 3 - GC-MS-based metabolomic analysis of plasma. Bar graphs show the relative abundance of metabolites in the healthy control (gray), and a GLUT1DS patient before (orange), and after (teal) L-fucose therapy that shift toward levels observed in the healthy control **(A)** and unchanged or shifted away from the healthy control **(B)** (**n = 1**).

5. Figure S 4

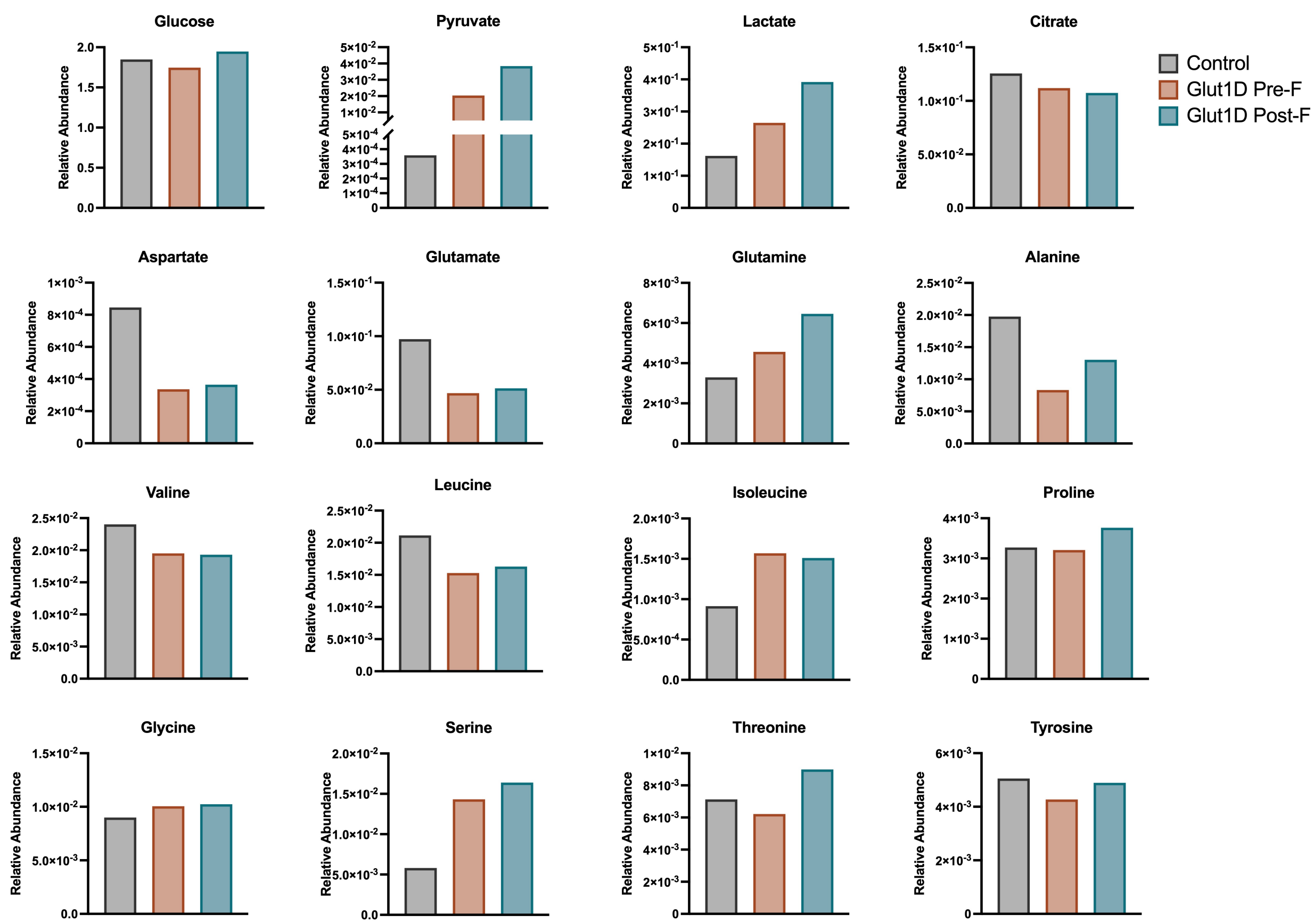

Figure S4 - GC–MS-based metabolomic analysis of CSF. Bar graphs show the relative abundance of metabolites in the healthy control (gray), and a patient with GLUT1 deficiency syndrome (Glut1DS) before (orange), and after (teal) L-fucose therapy (**n = 1**).
